# Acute Appendicitis Associated With Multisystem Inflammatory Syndrome in Children

**DOI:** 10.7759/cureus.15893

**Published:** 2021-06-24

**Authors:** Meghan E Hofto, Erinn O Schmit, Meenu Sharma, Nichole Samuy

**Affiliations:** 1 Pediatrics, Division of Pediatric Hospital Medicine, University of Alabama at Birmingham School of Medicine, Birmingham, USA

**Keywords:** sars-cov-2, multi-system inflammatory syndrome in children (mis-c), acute appendicitis, children, gastrointestinal symptoms

## Abstract

Multisystem inflammatory syndrome in children (MIS-C) has been reported to cause significant gastrointestinal symptoms. In this case series, we present four patients with MIS-C with documented acute appendicitis, which strengthens the association between SARS-CoV-2, MIS-C, and acute appendicitis.

## Introduction

Multisystem inflammatory syndrome in children (MIS-C) is defined by the Centers for Disease Control and Prevention (CDC) as “fever (≥38°C for more than 24 hours), laboratory evidence of inflammation, and evidence of clinically severe illness requiring hospitalization, with multisystem (≥2) organ involvement (cardiac, renal, respiratory, hematologic, gastrointestinal, dermatologic, or neurological)” associated with a current, suspected, or recent SARS-CoV-2 infection; to meet the case definition, there can be no other plausible diagnosis [1]. Some associated laboratory findings may include elevated C-reactive protein, elevated erythrocyte sedimentation rate, fibrinogen, d-dimer, ferritin, lactic acid dehydrogenase, and low albumin [1]. Gastrointestinal symptoms, including abdominal pain, vomiting, and diarrhea, have been some of the most common presenting symptoms in children with both MIS-C and novel Coronavirus Disease 2019 (COVID-19) [1-7]. Acute appendicitis is also associated with abdominal pain, vomiting, anorexia, fever, and elevated inflammatory markers, and it may be triggered by common viruses, including SARS-CoV-2 [8-10]. MIS-C has been noted in the literature to be an occasional mimicker of acute appendicitis, but little has been reported about acute appendicitis confirmed by pathology occurring concomitantly with MIS-C [11,12]. Here we present four patients with acute appendicitis temporally associated with MIS-C.

## Case presentation

Case 1

A 16-year-old white male with past medical history of hidradenitis suppurativa presented with three days of fever and abdominal pain and an episode of syncope to an outside hospital emergency department. His symptoms began with fever, and right lower quadrant abdominal pain began the next day. He presented to the emergency department on the third day of illness and was admitted. On hospital day 1, computed tomography of his abdomen revealed a low-lying cecum and peri-rectal inflammation. He was evaluated by general surgery and underwent a laparoscopic appendectomy with pathology confirmatory for acute appendicitis. Post-operatively, he remained persistently febrile. On hospital day 6, he developed hypotension, requiring transfer to pediatric intensive care unit and vasopressors. On hospital day 7, he was transferred to the local tertiary-care children’s hospital pediatric intensive care unit on dopamine and milrinone. Laboratory results were consistent with myocarditis, and an echocardiogram demonstrated diffuse coronary dilatation (Table 1). Physical examination on arrival to the tertiary care intensive care unit revealed a grade III/VI systolic ejection murmur over the lower left sternal border, a gallop, and tenderness to palpation in the right lower quadrant without guarding. He was empirically started on broad-spectrum antibiotics, including doxycycline for concern for possible rickettsial disease; all except doxycycline were discontinued with negative blood cultures and a negative test for microbial cell-free DNA in the blood. Doxycycline was continued empirically given prevalence of Rocky Mountain Spotted Fever in the area. Due to the echocardiographic findings of diffuse coronary dilation, he was discussed with cardiology with concern for atypical Kawasaki disease. He was treated with intravenous immunoglobulin (IVIG) and aspirin on hospital day 7 (day 9 of fevers) with rapid defervescence and improvement in his blood pressures. Following IVIG completion, he became afebrile and abdominal pain also resolved by the following morning. Cardiac labs including troponin, brain natriuretic peptide, and electrocardiogram were trended until normalization occurred, at which point he was discharged home on aspirin therapy. A SARS-CoV-2 PCR was performed after transfer (hospital day 8) and was negative, and SARS-CoV-2 serologies were not performed during his admission. This patient was admitted prior to the official CDC definition of MIS-C requiring current or recent SARS-CoV-2 infection, but local experts felt his presentation was consistent with the then-current case definition for Pediatric Inflammatory Multisystem Syndrome or MIS-C.

**Table 1:** Initial inflammatory labs and cardiac testing consistent with Multisystem Inflammatory Syndrome in Children. Values in bold are considered abnormal*; normal values for our laboratory are listed with each test**. *MIS-C is associated with elevated C-reactive protein (CRP), erythrocyte sedimentation rate (ESR), fibrinogen, procalcitonin, d-dimer, ferritin, lactic acid dehydrogenase (LDH), or interleukin 6 (IL-6), elevated neutrophils, reduced lymphocytes and low albumin. **Normal values can significantly vary by age; the value listed in parentheses by the laboratory test name is the normal value for all patients, while the values listed in parentheses under each case are the age-based normal for that test.

	Patient 1	Patient 2	Patient 3	Patient 4
SARS-CoV-2 PCR	Negative	Negative	Positive	Positive
SARS-CoV-2 IgG	--	Positive	Positive	Positive
White Blood Cell Count	13.25 (3.8-9.8 x 10^3^/uL)	3.4 (3.8-9.8 x 10^3^/uL)	15.79 (6.5-13 x 10^3^/uL)	15 (4.19-9.43 x 10^3^/uL)
Neutrophil	81.1 (32.5-74.7%)	68.6 (32.5-74.7%)	70.0 (16.9-74%)	90.4 (39.0-73.6%)
Lymphocytes	11.2 (16.4-52.7%)	16.0 (16.4-52.7%)	17.9 (27.4-79.9%)	4.6 (18.2-49.8%)
Hemoglobin	8.8 (11-14.5 g/dL)	12.1 (11-14.5 g/dL)	9.9 (10.2-12.7 g/dL)	9.4 (10.8-13.3 g/dL)
Platelet Count (140-440 x 10^3^/uL)	25.7	145	117	256
Erythrocyte Sedimentation Rate (0-15 mm/h)	76	26	37	83
C-Reactive Protein (0-0.5 mg/dL)	15.7	15.5	28.2	30.6
Lactate Dehydrogenase Level	--	300 (170-283 U/L)	401 (192-321 U/L)	209 (130-250 U/L)
Troponin 1 (< 0.05 ng/mL)	0.36	0.74	<0.01	0.04
Ferritin Level	517.2 (11.1-171.9 ng/mL)	196.5 (13.7-78.8 ng/mL)	515.9 (5.3-99.9 ng/mL)	472 (5.5-67.4 ng/mL)
D-Dimer FEU (<0.51 ug/mL)	--	10.2	18.66	6.39
Fibrinogen Level (156-400 mg/dL)	--	479	411	767
Brain Natriuretic Peptide (7-21 pg/mL)	1012.2	161.8	38.8	886.6
INR (0.9-1.2)	1.2	1.3	1.8	1.4
Albumin	2.3 (4.1-5.1 g/dL)	3.9 (4.1-4.8 g/dL)	2.3 (3.8-4.7 g/dL)	2.8 (4.0-4.9 g/dL)
Sodium (138-145 mmol/L)	140	134	136	137
Creatinine	1.42 (0.62-1.08 mg/dL)	0.69 (0.45-0.81 mg/dL)	0.26 (0.1-0.36 mg/dL)	0.79 (0.49-0.84 mg/dL)
Alanine Transaminase Level (9-24 U/L)	20.1	20.8	15.7	33.6
Aspartate Aminotransferase Level (14-35 U/L)	36	52	36	31
Electrocardiogram	Sinus tachycardia; borderline left ventricular hypertrophy	Sinus tachycardia	Sinus tachycardia	Sinus tachycardia
Echocardiogram	Mild diffuse dilation of all coronary arteries	Normal cardiac anatomy and function	Small pericardial effusion	Low normal left ventricular systolic function
Treatment	IVIG + aspirin	IVIG 2 g/kg + aspirin	IVIG 2 g/kg + steroids + aspirin	IVIG + steroids + aspirin

Case 2

A previously healthy 13-year-old Hispanic male presented to the emergency department with three days of right lower quadrant pain, nausea, vomiting and fever to 39.4°C; symptoms initially began with fever and progressed to worsening abdominal pain. History obtained on admission revealed a respiratory infection with anosmia and positive COVID-19 household contacts one month prior to presentation. Physical examination revealed an overall well-appearing male with abdominal tenderness to palpation in the right lower quadrant, without rebound tenderness. Abdominal ultrasound was suggestive of acute appendicitis with a possible small rupture without a surrounding abscess, and he was admitted for a laparoscopic appendectomy and received one dose of piperacillin/tazobactam pre-operatively. Pathology report was consistent with early acute appendicitis. His post-operative course was complicated by persistent fever up to 39.9°C and abdominal pain. Infectious studies, including multiplex viral polymerase chain reaction (PCR), rapid streptococcal antigen testing, and urinalysis, were negative. SARS-CoV-2 PCR testing was negative, but SARS-CoV-2 IgG antibody testing was positive. His labs were consistent with MIS-C (Table 1). He was given IVIG on the evening of hospital day 2 and low dose aspirin with improvement in his fever curve (fully defervesced on hospital day 4), abdominal pain, and inflammatory labs. He was discharged home on hospital day 5 with aspirin. He followed up outpatient in a multidisciplinary clinic for patients with SARS-CoV-2 infection and MIS-C with no further sequelae or cardiac involvement.

Case 3

**Figure 1:**
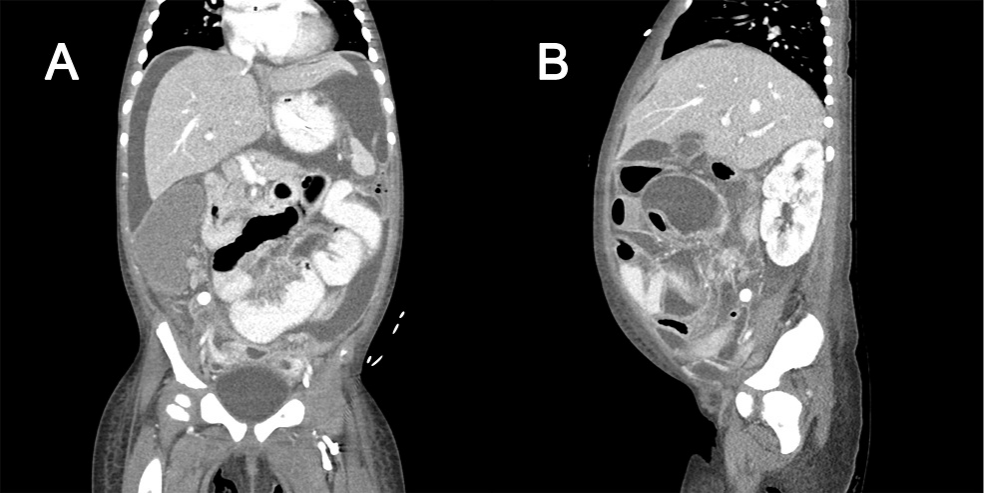
Computed tomography of the abdomen of Patient 3. Coronal plane image (A) demonstrating large amount of perihepatic intraperitoneal fluid and diffuse peritonitis. Sagittal plane image (B) demonstrating subdiaphragmatic fluid collections and appendicolith.

A 20-month-old female with no significant past medical history was admitted to a community hospital due to a one-day history of fever and dehydration and was found to have a positive PCR test for SARS-CoV-2. She was started on empiric ceftriaxone and later azithromycin for presumed pneumonia. On hospital day 7 (day 8 of fever), the patient was transferred to the local tertiary-care children’s hospital due to persistent fever and diffuse abdominal pain, distention, and diarrhea that began two days prior to transfer. Physical examination revealed an ill-appearing female with a diffusely tender, markedly distended, and firm abdomen with hypoactive bowel sounds. She had no rash, no conjunctival injection, no extremity swelling, and no lymphadenopathy. Abdominal radiography performed on the day of transfer showed gas-distended loops of bowel with air-fluid levels and no free intraperitoneal air, likely ileus. Laboratory findings, electrocardiogram, and echocardiogram were consistent with a diagnosis of MIS-C (Table 1). The patient was treated with IVIG and aspirin the day of transfer. The morning after transfer, she was noted to have worsening abdominal distention and continued fever despite acetaminophen. Repeat abdominal radiography was obtained and was again consistent with ileus. A nasogastric tube was placed, empiric piperacillin/tazobactam was started, and steroids were initiated for continued treatment of MIS-C. The patient demonstrated clinical improvement following these interventions. On hospital day 11, the patient demonstrated acute clinical deterioration with significant increase in abdominal girth, worsening tenderness, and near-absent bowel sounds. Urgent computed tomography of the abdomen was obtained and demonstrated perforated appendicitis with discontinuous appearance of the appendix, peritonitis, intraperitoneal fluid, and free intraperitoneal air (Figure 1). Abdominal drains were placed by interventional radiology on hospital days 12, 15, and 20 for drainage of multiple intra-abdominal abscesses. She defervesced on hospital day 18. She was treated with intravenous piperacillin/tazobactam for a total of 18 days and was subsequently transitioned to oral ciprofloxacin and metronidazole upon abdominal drain removal on the day of discharge. She was discharged home on hospital day 27 on the same antibiotic regimen, steroids, and aspirin.

Case 4

A previously healthy 17-year-old black female presented to an outside hospital for evaluation of diffuse abdominal pain and fevers up to 38.8°C preceded by two days of nausea, vomiting, and diarrhea. The patient had positive COVID-19 household contacts one month prior to presentation. She was initially diagnosed with appendicitis and underwent laparoscopic appendectomy. The pathology report confirmed a mild acute appendicitis. Her hospital course was complicated by development of acute hypoxic respiratory failure and persistent fever and abdominal pain. A computed tomography of her chest, abdomen, and pelvis revealed bilateral pleural effusions and intestinal ileus. The patient was transferred to a tertiary children’s hospital. Physical exam on transfer revealed an ill-appearing female with respiratory distress, cool extremities, delayed capillary refill, and diffusely tender abdomen with distension and guarding; vital signs were significant for fever, hypotension, and tachycardia. Empiric antibiotics were started including vancomycin, ceftriaxone, and metronidazole; vancomycin and ceftriaxone were continued for five days. Repeat computed tomography of the chest, abdomen, and pelvis continued to show bilateral pleural effusions with scattered ground-glass opacities and post-operative ileus. Both SARS-CoV-2 PCR and SARS-CoV-2 IgG antibody tests were positive. Her laboratory findings were consistent with MIS-C (Table 1). Her electrocardiogram showed sinus tachycardia, and an echocardiogram revealed low normal left ventricular systolic function. The patient received IVIG on the morning of hospital day 5 and was started on steroids and low-dose aspirin. The patient’s fever resolved on hospital day 5, and she remained afebrile for the remainder of her hospitalization. She continued to endorse diffuse abdominal pain to palpation without rebound or guarding which gradually improved, and the patient endorsed minimal abdominal pain at discharge. She was discharged home on steroids and aspirin.

## Discussion

All four of the patients in this case series had evidence of acute appendicitis, with pathologic confirmation and surgical management. All four also met criteria for MIS-C. While initial imaging for three patients demonstrated possible appendicitis, the patient in case 3 did not have radiological evidence of appendicitis until after her illness had progressed to perforation. Gastrointestinal symptoms are one of the most common presenting symptoms of MIS-C and have been found in up to 90% of patients [2-7,10]. Children with SARS-CoV-2 have previously been found to have atypical appendicitis associated with terminal ileitis; however, unlike the patients reported here, those patients did not require surgical management [10]. Abdominal imaging should be considered in all MIS-C patients, especially if pain continues or worsens after initial therapy for MIS-C.

Three of the reported patients were initially admitted with a diagnosis of appendicitis and had an appendectomy prior to further diagnosis of MIS-C; the fourth had an initial diagnosis of MIS-C and progressed to appendicitis. As surgeons commonly manage patients with appendicitis, they should be familiar with the diagnostic criteria for MIS-C in order to identify suspected MIS-C patients earlier and facilitate consultation with MIS-C experts and initiation of treatment as needed. Pediatricians should also be aware of an association between MIS-C and appendicitis to consider appendicitis as a possible cause of abdominal pain in these patients and have a lower threshold for imaging and/or surgical consultation.

All four patients empirically received broad-spectrum antibiotics prior to the final diagnosis given the initial presentation. The third patient required prolonged antibiotics for perforated appendicitis with associated intra-abdominal abscess. Non-operative management of acute uncomplicated appendicitis with antibiotics alone has been shown to be successful for many children, and empiric antibiotics are routinely recommended for children with acute appendicitis [13,14]. Given the potential morbidity and mortality of untreated appendicitis, clinicians could consider broad-spectrum antibiotics to empirically cover abdominal pathogens in patients with severe MIS-C and associated abdominal pain, particularly if symptoms do not resolve as expected with initial treatment.

## Conclusions

This case series further strengthens the association between SARS-CoV-2, MIS-C, and acute appendicitis. This overall emphasizes the need to maintain a broad differential diagnosis, particularly in patients with abdominal pain and gastrointestinal symptoms with recent SARS-CoV-2 infection. Pediatricians and surgeons should be aware of the possibility of MIS-C in patients with appendicitis, or appendicitis in patients with MIS-C, particularly if they do not defervesce and improve as expected following standard treatment.
